# Supplementary figures and images for: Efficient sex separation by exploiting differential alternative splicing of a dominant marker in Aedes aegypti
Source: PLoS Genet. 2023 Nov 27;19(11):e1011065. doi: 10.1371/journal.pgen.1011065 (PMC10703412; doi:10.1371/journal.pgen.1011065)

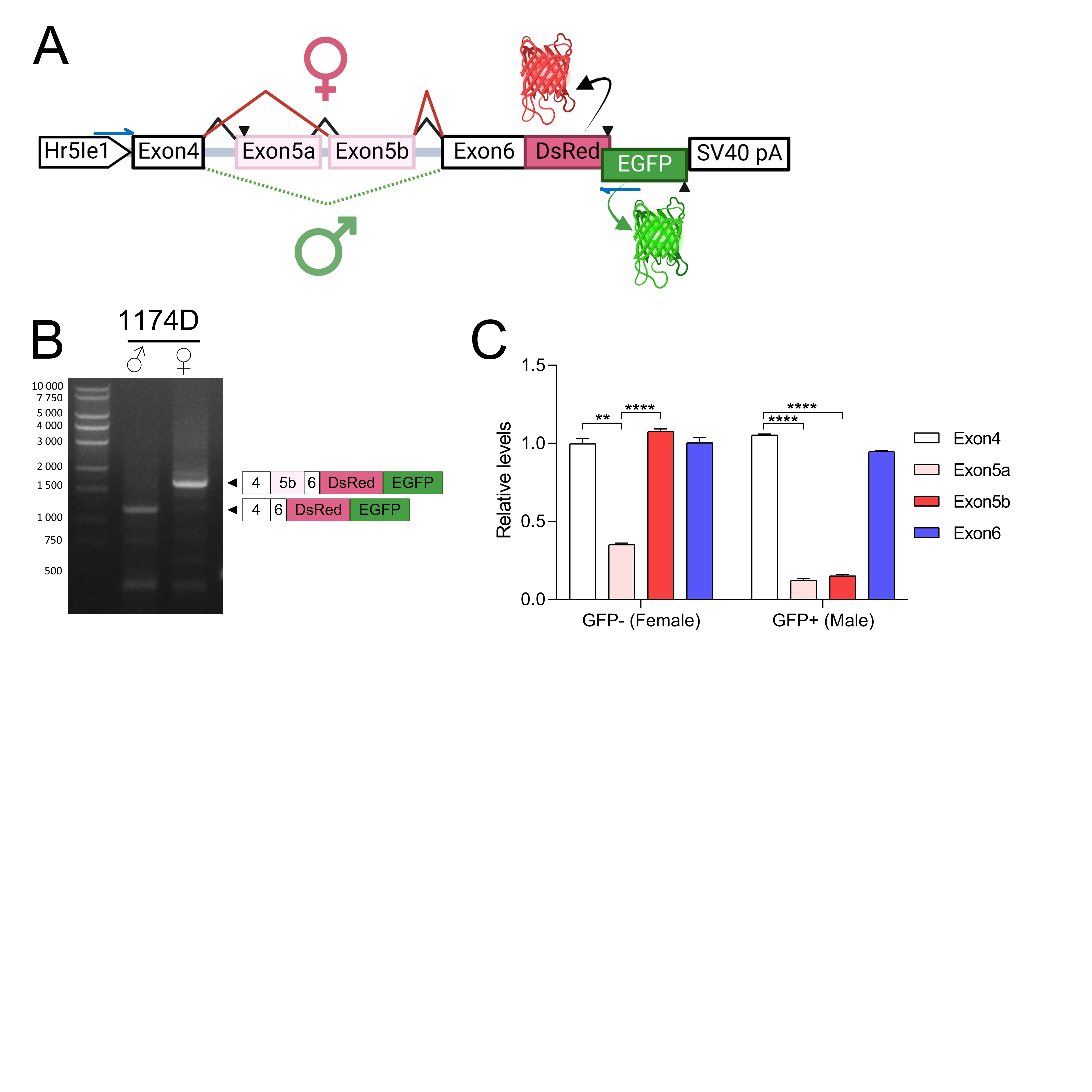

Supplement: S1 Fig — Fifty GFP-positive larvae and fifty GFP-negative larvae at the L1 stage were carefully sorted, and total RNA was extracted from each group. To determine the splicing patterns, RT-PCR was performed using specific primers targeting the 3’ end of the Hr5Ie1 promoter sequence and the 5’ end of the EGFP coding sequence. (A) The relative locations of the primer target sites are indicated by blue arrows. (B) The PCR products were subsequently purified and subjected to sequencing in order to validate the splicing junctions. The resulting splicing patterns are depicted in the right panel. (C) The relative levels of non-sex-specifically regulated exons (exon4 and exon6) and female-specific exons (exon5a, exon5b) of SEPARATOR were determined through RNA sequencing (RNAseq) analysis. The FPKM (fragments per kilobase per million mapped reads) values of each exon were normalized using the average FPKM of the non-sex-specifically regulated exons (exon4 and exon6). The bar plot displays the means and ± SD (standard deviation) for triple biological replicates. Statistical significance of mean differences was assessed using a Tukey’s multiple comparisons test, with p-values denoted as follows: p < 0.01** and p < 0.0001****. Figure was created using BioRender. (TIF) [file pgen.1011065.s001.TIF]

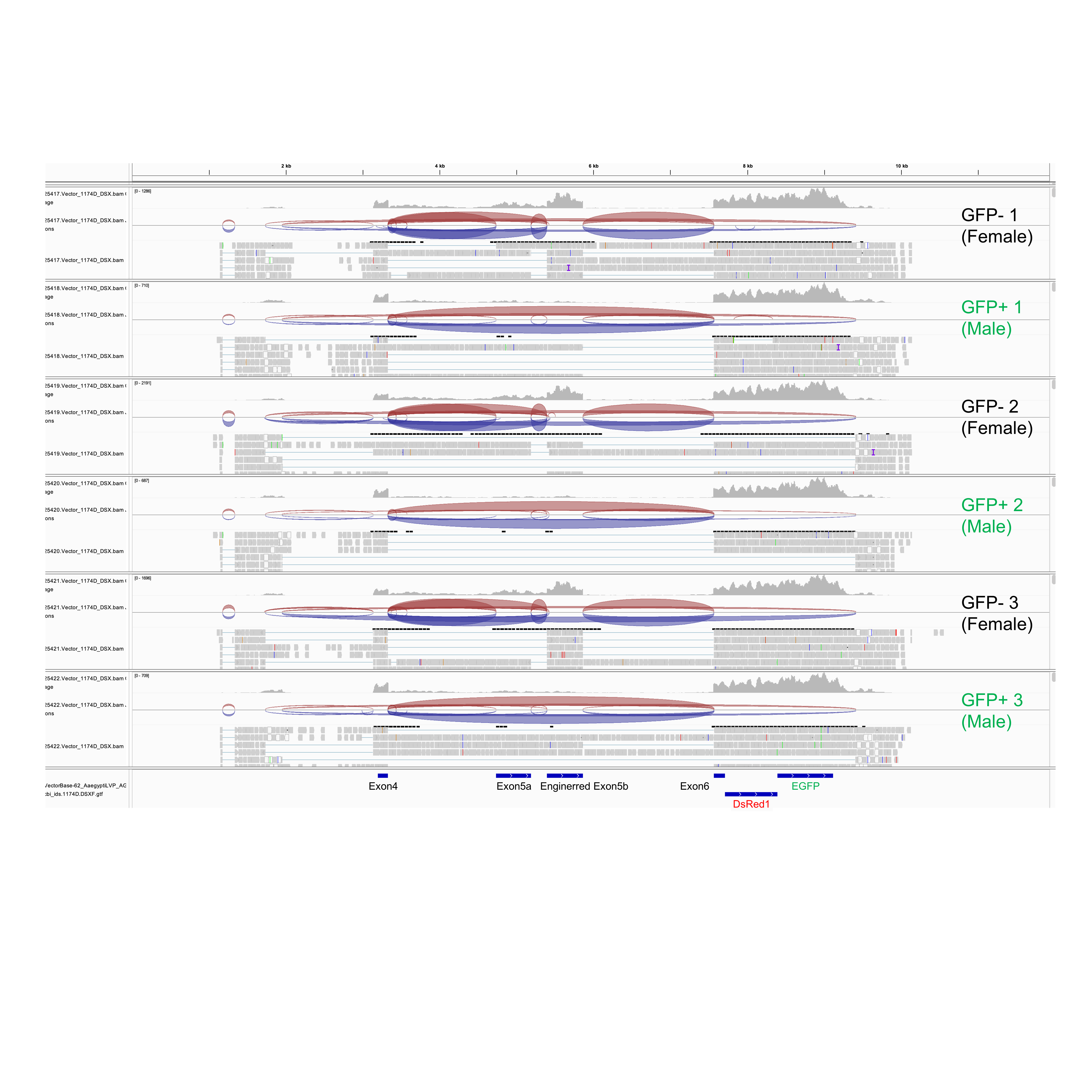

Supplement: S2 Fig — The splicing patterns of SEPARATOR were verified through RNAseq analysis in both GFP-positive and GFP-negative mosquitoes, with triple biological replicates for each condition. The RNAseq reads for the different genotypes were aligned, and the location of exons is indicated at the bottom in blue. (TIF) [file pgen.1011065.s002.TIF]

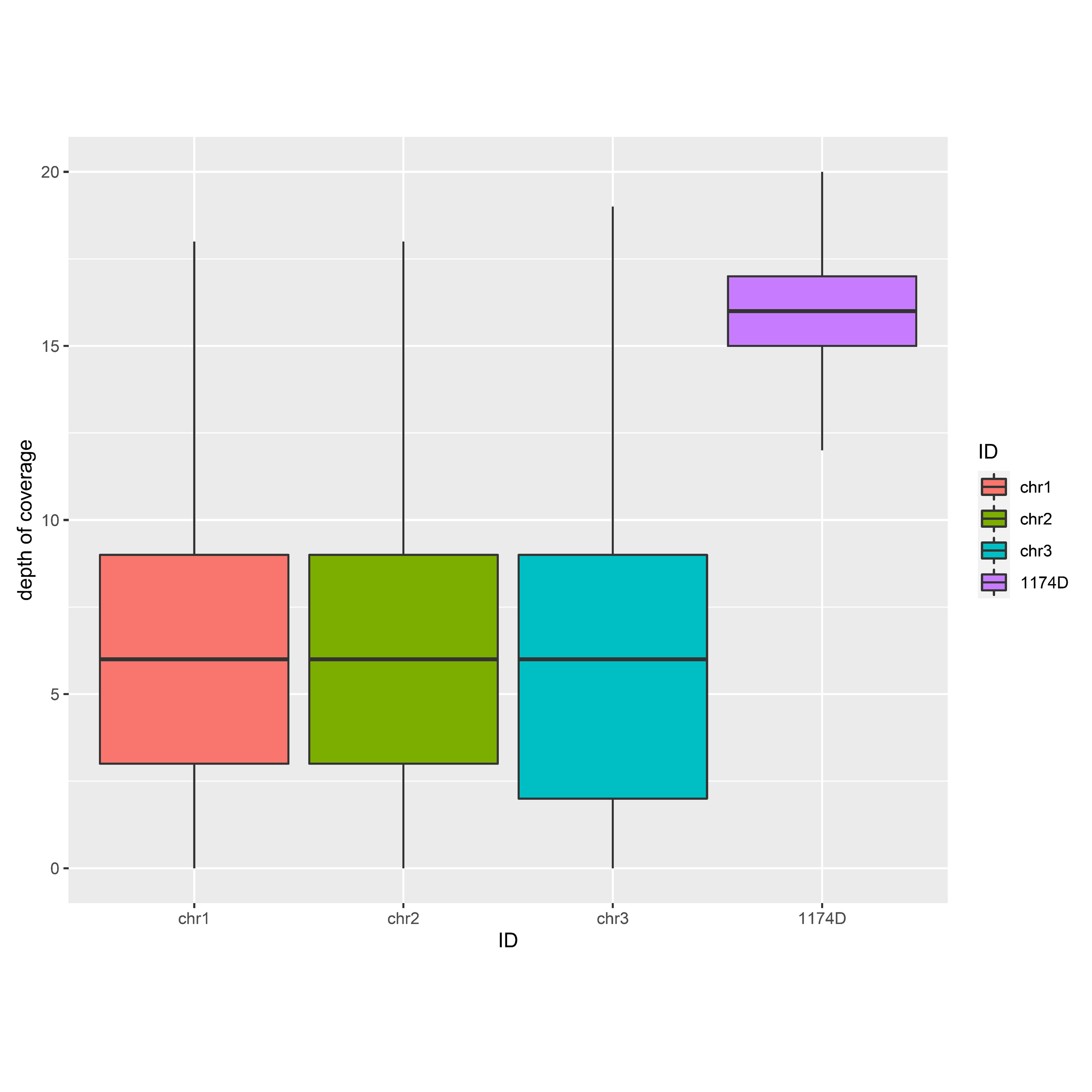

Supplement: S3 Fig — A standard box plot is used to illustrate the coverage distributions of three chromosomes (Chr1, Chr2 and Chr3) and the SEPARATOR transgenes (1174D) in SEPARATOR mosquitoes. The center line represents the median, while the first and third quartiles define the boundaries of the box. The upper and lower whiskers extend from the box to the highest and lowest observed values, respectively, but no further than 1.5 times the Interquartile Range (IQR) from the box. Based on the sequencing depths, the coverage for chromosomes 1, 2, and 3 were 6.31, 6.30, and 6.08, respectively, while the coverage for the SEPARATOR transgenes was 16.14. From the coverage analysis, it suggests that the SEPARATOR transgene (1174D) is present in three copies. (TIF) [file pgen.1011065.s003.TIF]

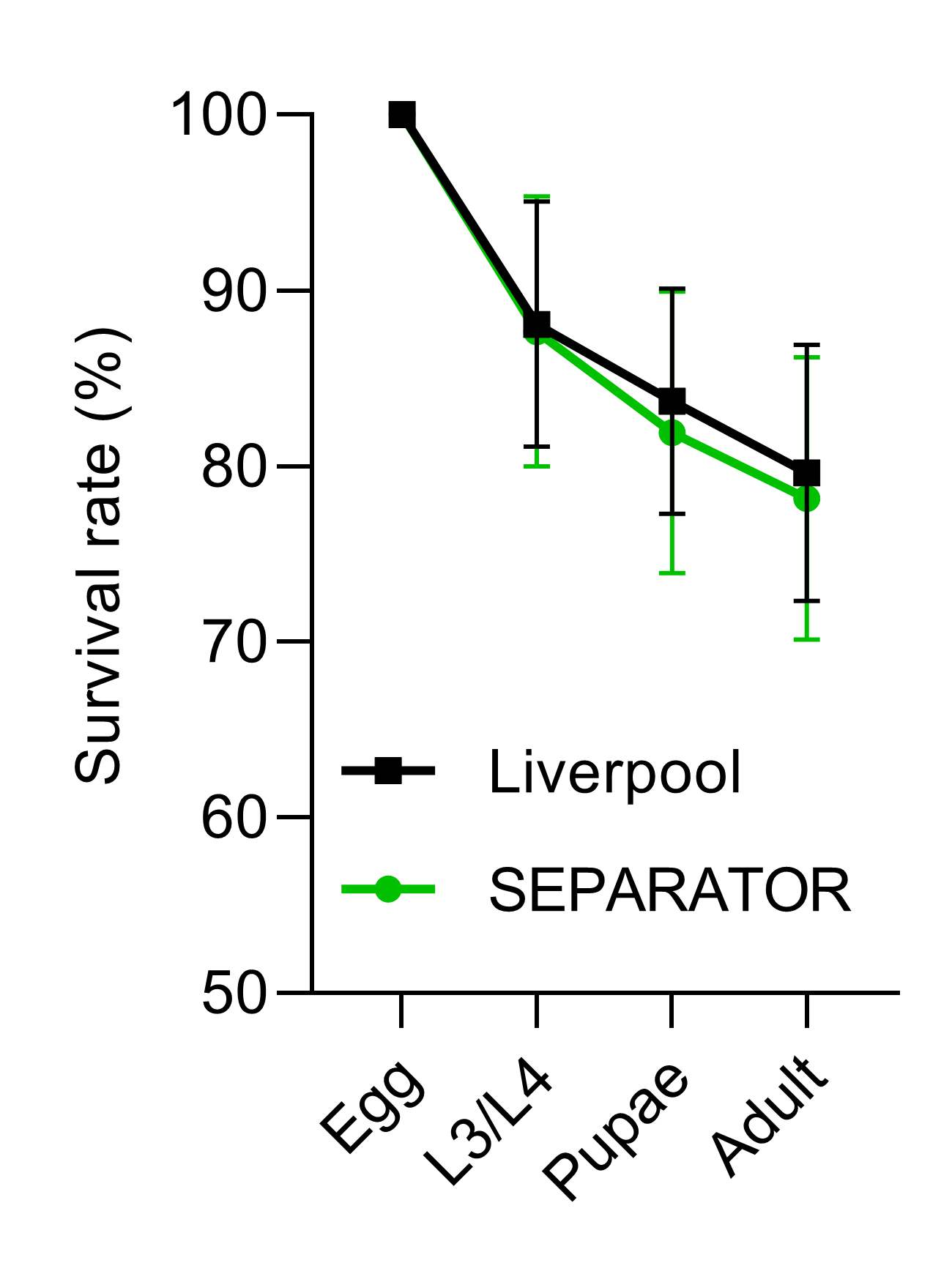

Supplement: S4 Fig — Eggs from female mosquitoes of the wild-type (Liverpool) and SEPARATOR strains were individually collected and quantified. The hatched larvae were cultured, and their numbers were counted at the L3 and L4 larval, pupal, and adult stages. (TIF) [file pgen.1011065.s004.tif]

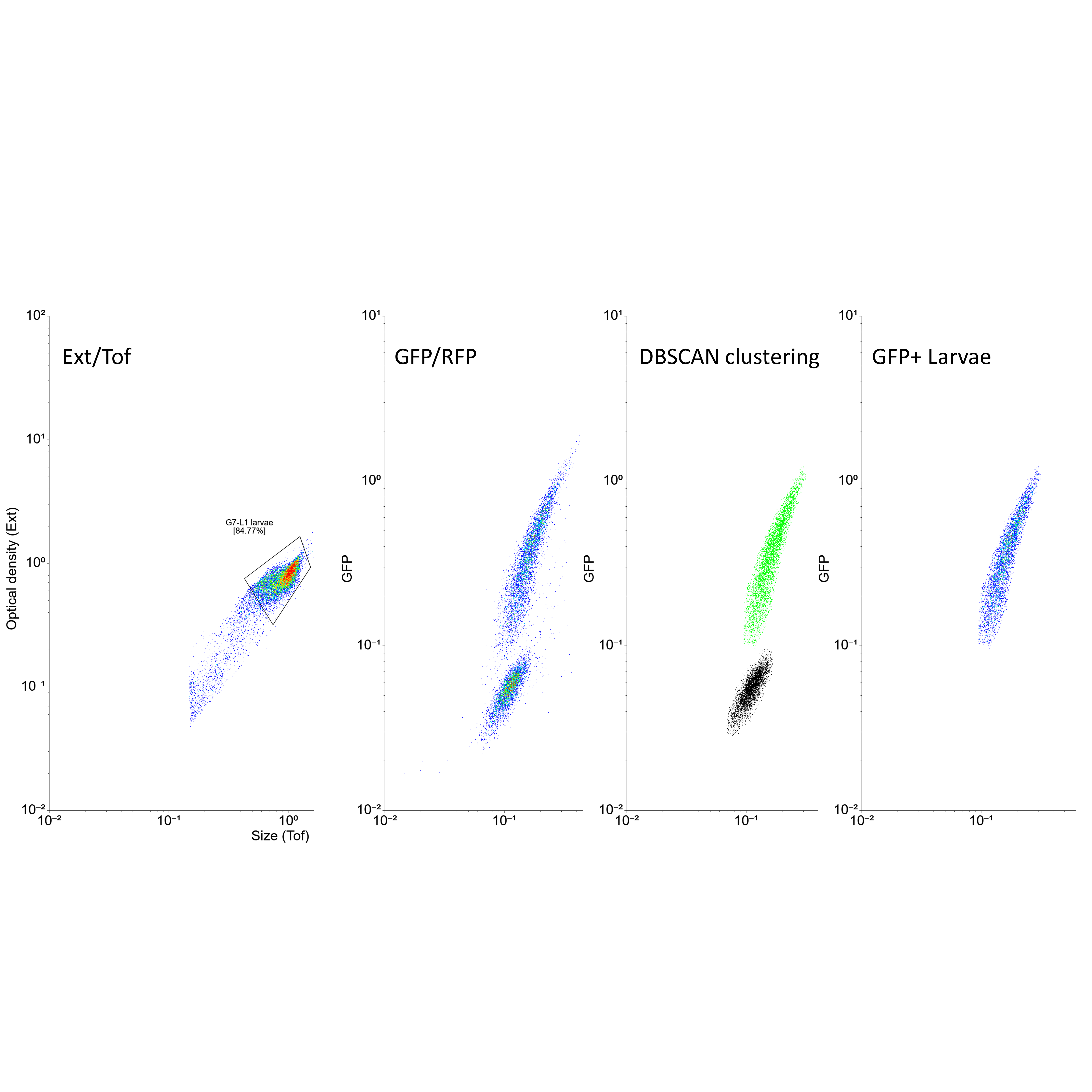

Supplement: S5 Fig — The COPAS raw data was initially filtered for the larvae using size and optical density criteria (Ext/Tof). Next, the particles that exhibited fluorescence (GFP/RFP) were gated. Subsequently, DBSCAN clustering was used to automatically cluster and denoise the data. Finally, the larvae that were GFP-positive were selected. (TIF) [file pgen.1011065.s005.TIF]

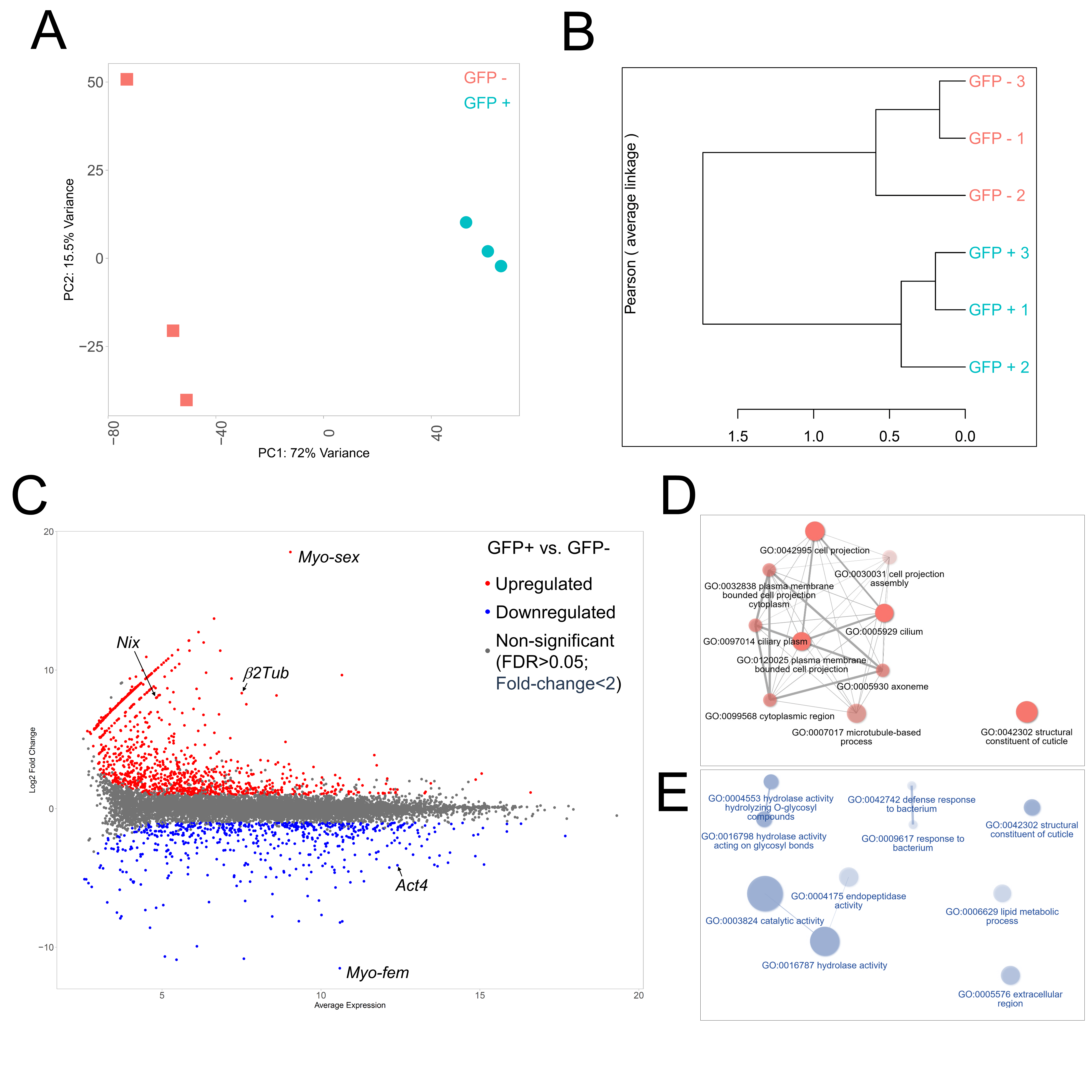

Supplement: S6 Fig — (A) PCA analysis and (B) hierarchical clustering of six samples used for RNA sequencing. (C) MA-plots were generated to visualize the differential expression patterns between GFP-positive and GFP-negative larvae at the L1 stage in SEPARATOR mosquitoes. In the plot, significantly upregulated (male-enriched) genes (FDR < 0.05 and fold-change > 2) are indicated by red dots, significantly downregulated (female-enriched) genes (FDR < 0.05 and fold-change > 2) are indicated by blue dots, and non-significantly differentially expressed genes are represented by gray dots (FDR > 0.05 or fold-change < 2). Additionally, five well-known sex-enriched genes were marked in the plot. A network visualization was created to illustrate the relationship among enriched Gene Ontology (GO) terms for the upregulated (D) and downregulated (E) genes. (TIF) [file pgen.1011065.s006.TIF]

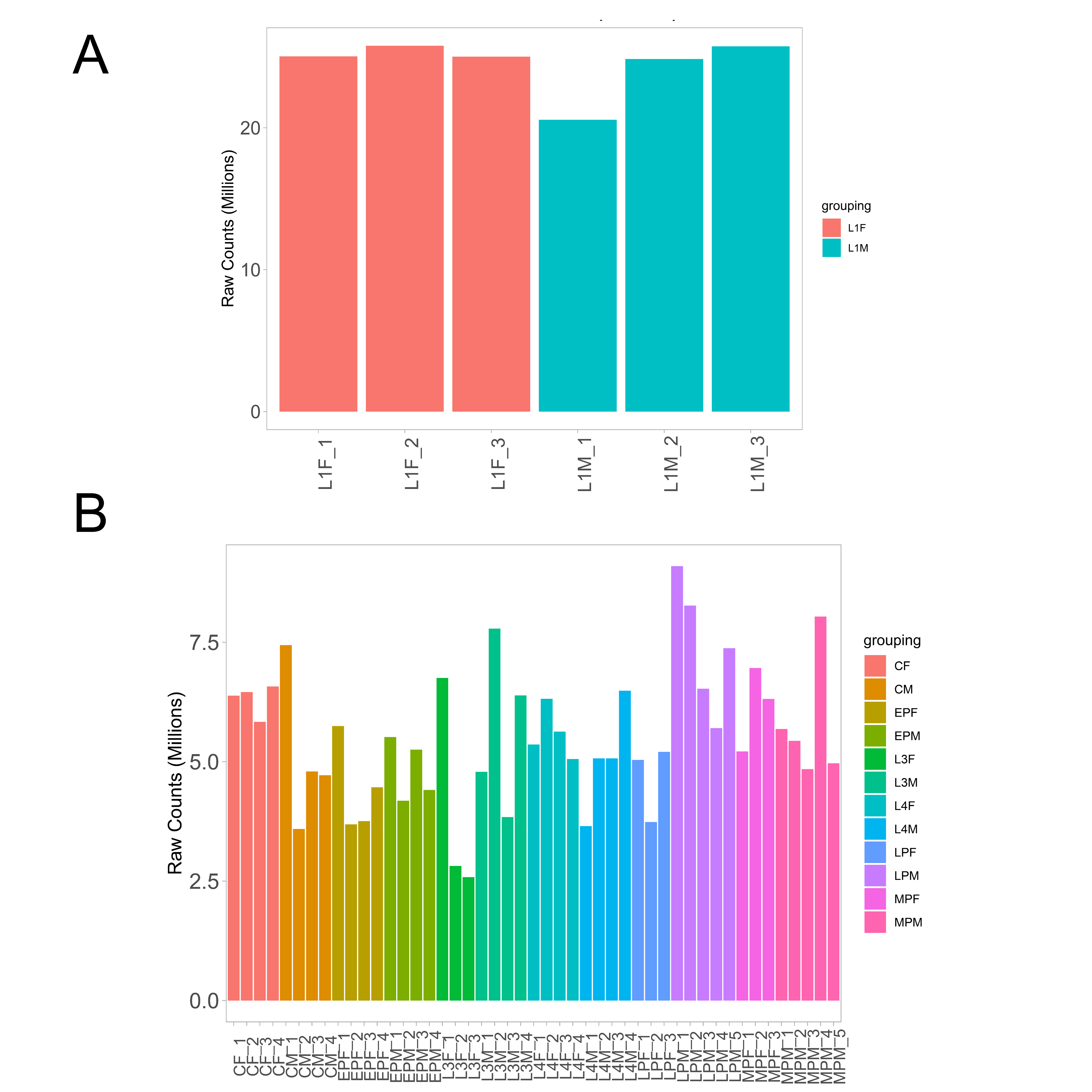

Supplement: S7 Fig — In the transcriptome comparison analysis, we employed GFP-positive (Male, L1M) and GFP-negative (Female, L1F) larvae at the L1 stage from SEPARATOR mosquitoes. Additionally, we utilized larvae at the L3 (L3M and L3F) and L4 (L4M and L4F) stages, as well as early pupae (EPM and EPF), mid pupae (MPM and MPF), late pupae (LPM and LPF), and carcass of adult mosquitoes (CM and CF) from Matthews’s RNA-seq datasets. The sequencing depth of the RNA-Seq data was achieved through the utilization of an integrated web application known as iDEP. (TIF) [file pgen.1011065.s007.TIF]

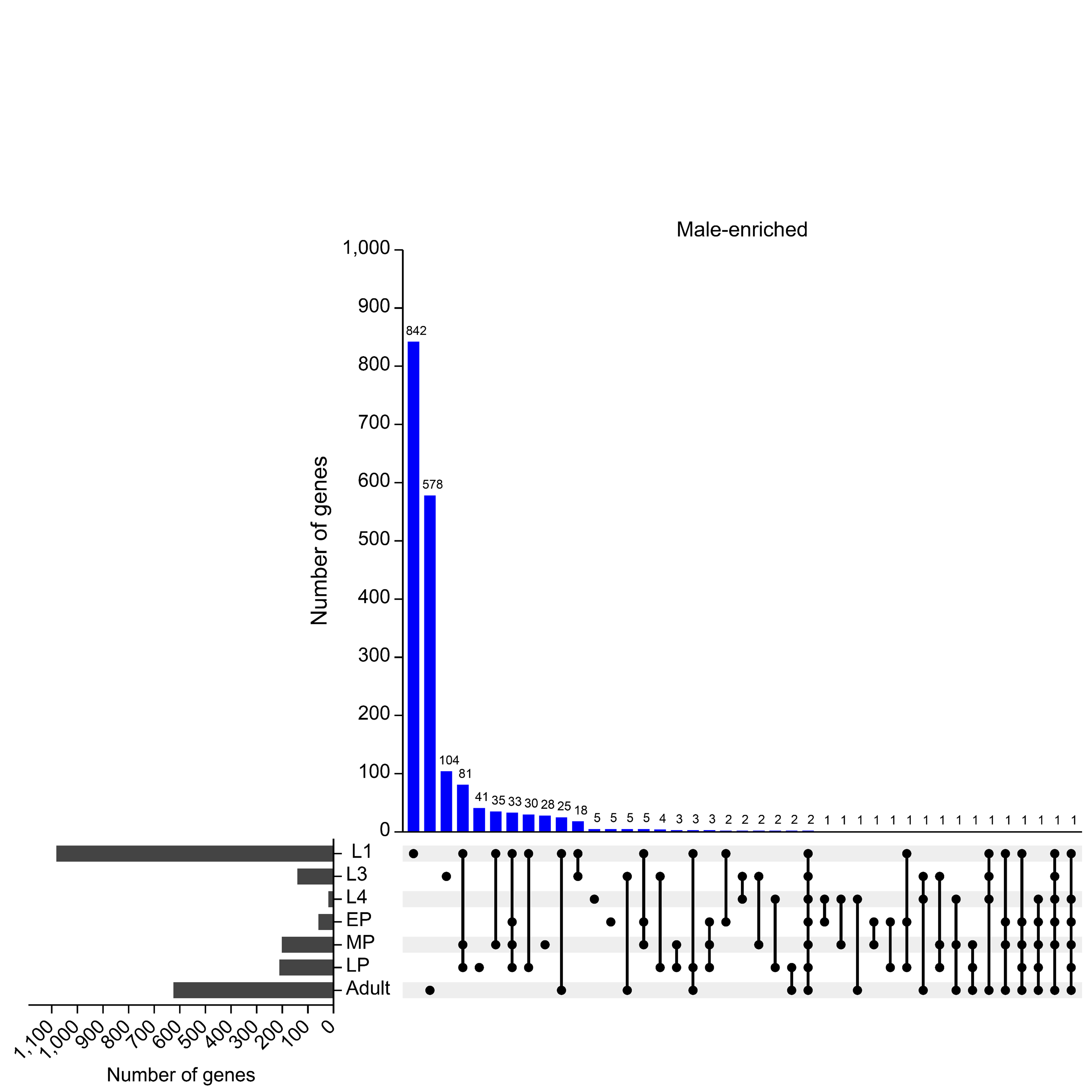

Supplement: S8 Fig — In our transcriptome comparison analysis, we included L1 stage larvae from SEPARATOR mosquitoes. Furthermore, we incorporated L3 and L4 stage larvae, along with early pupae (EP), mid pupae (MP), late pupae (LP), and adult mosquito carcass (Adult) from Matthews’s RNA-seq datasets. The correlation of male-enriched genes in this analysis was visualized using an UpSet plot, facilitated by the integrated web application iDEP. (TIF) [file pgen.1011065.s008.TIF]

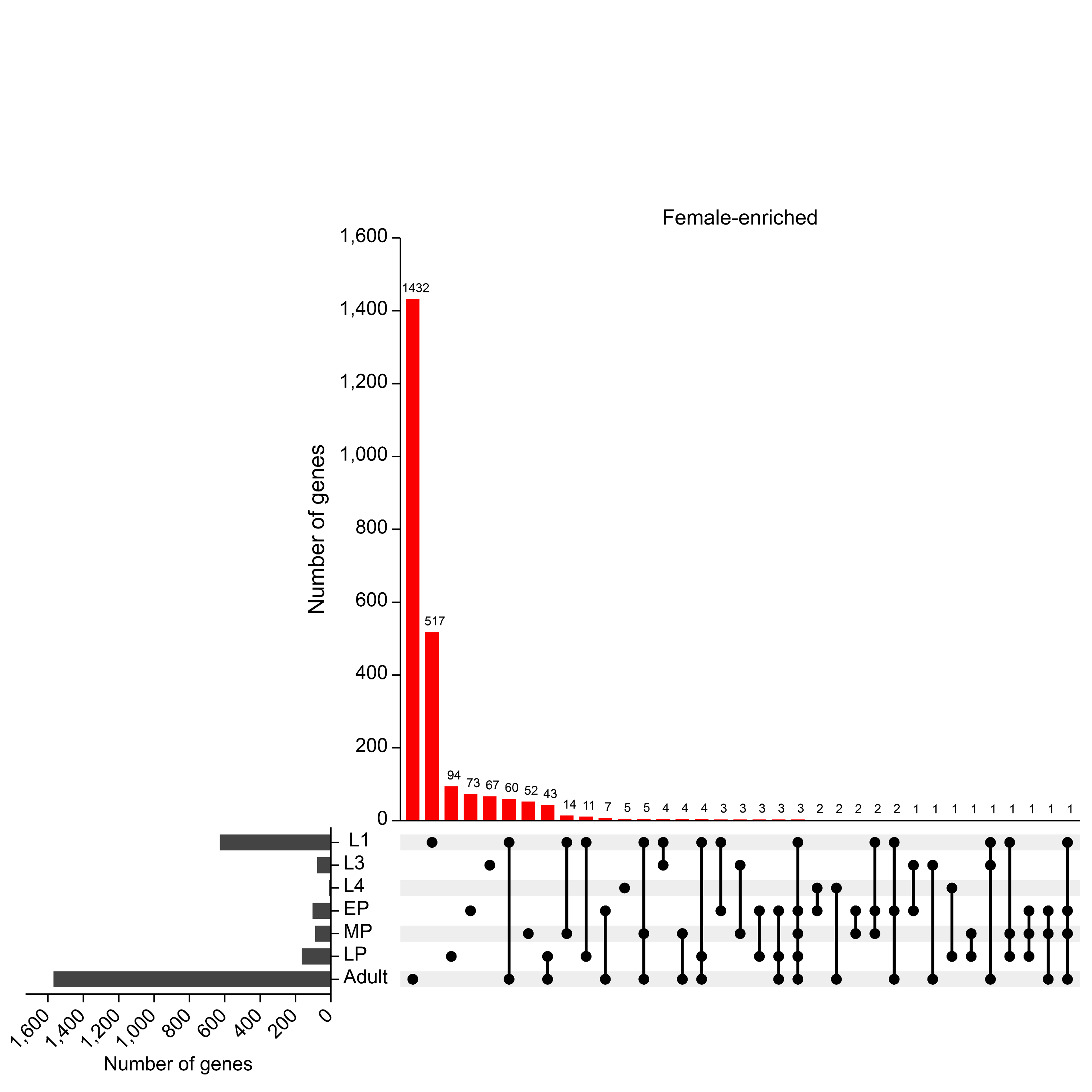

Supplement: S9 Fig — In our transcriptome comparison analysis, we included L1 stage larvae from SEPARATOR mosquitoes. Furthermore, we incorporated L3 and L4 stage larvae, along with early pupae (EP), mid pupae (MP), late pupae (LP), and adult mosquito carcass (Adult) from Matthews’s RNA-seq datasets. The correlation of female-enriched genes in this analysis was visualized using an UpSet plot, facilitated by the integrated web application iDEP. (TIF) [file pgen.1011065.s009.TIF]

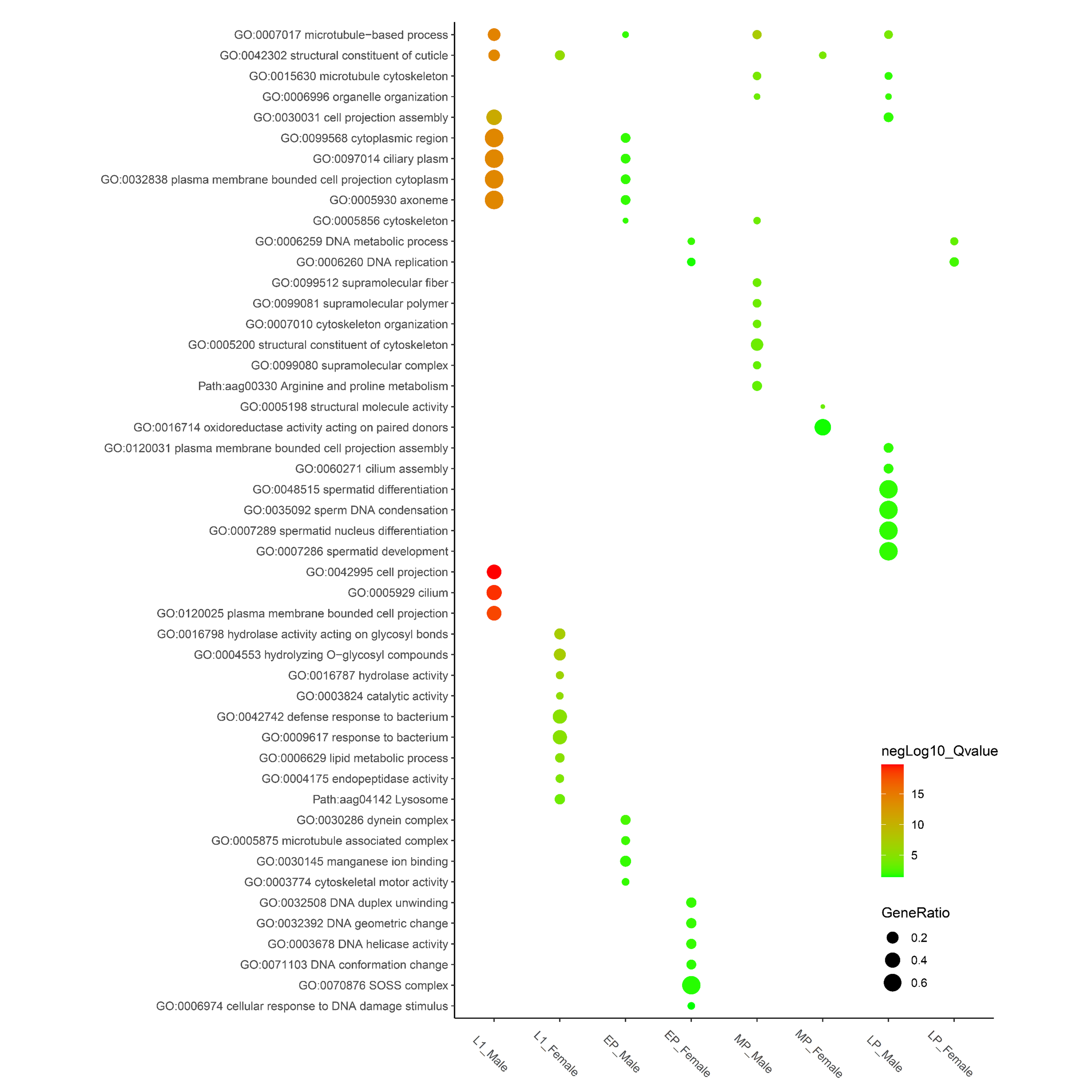

Supplement: S10 Fig — In our transcriptome comparison analysis, we included L1 stage larvae from SEPARATOR mosquitoes. Furthermore, we incorporated early pupae (EP), mid pupae (MP), and late pupae (LP), from Matthews’s RNA-seq datasets. The correlation of GO terms in sex-enriched genes within this analysis was identified and facilitated by the integrated web application iDEP. (TIF) [file pgen.1011065.s010.TIF]

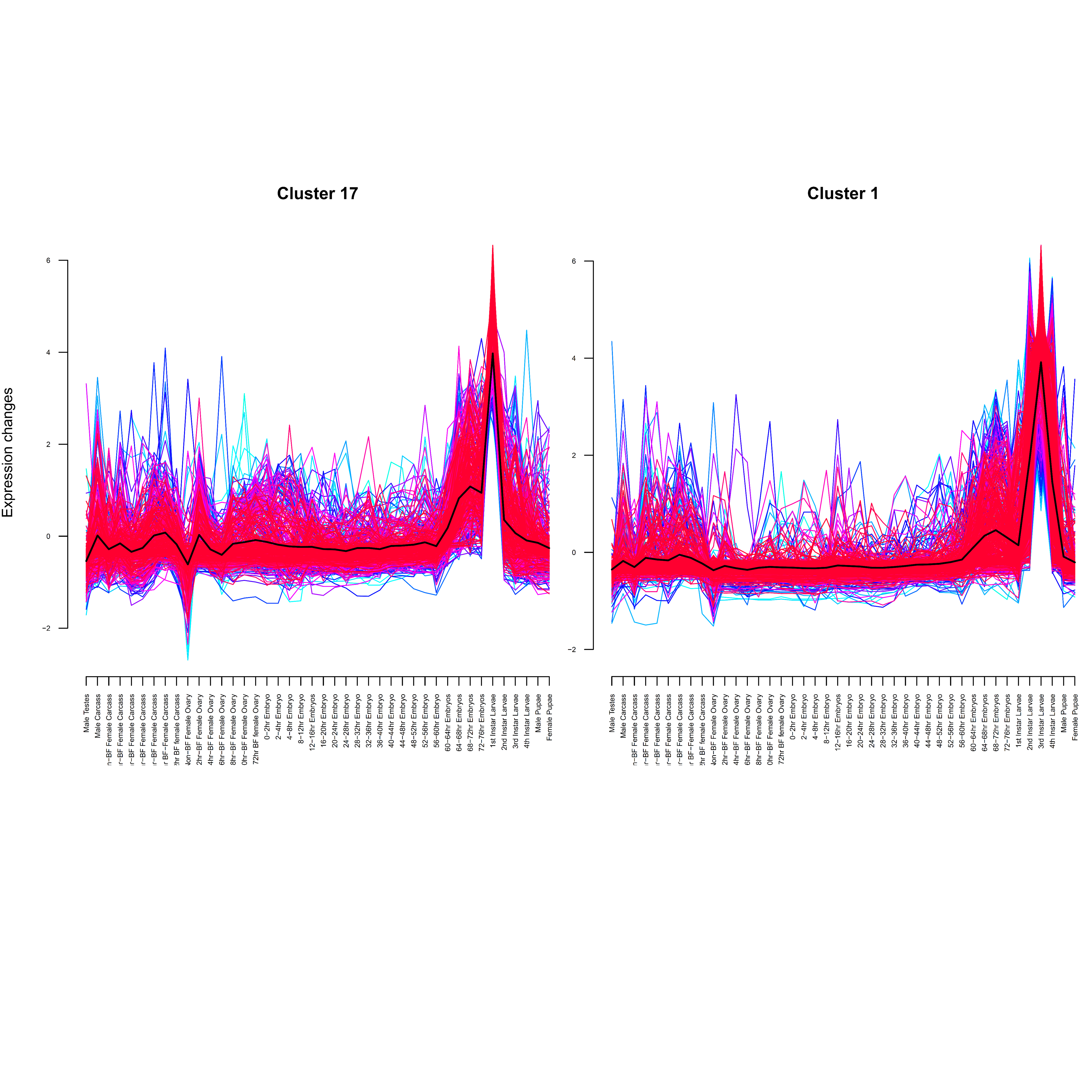

Supplement: S11 Fig — Through mfuzz clustering analysis using comprehensive developmental stage data from a previous study, specific genes associated with either L1 or L2-L4 stages were identified. Notably, cluster 17 predominantly consisted of genes expressed in L1, while cluster 1 exhibited gene expression primarily in L2-L4 stages. (TIF) [file pgen.1011065.s011.TIF]
